# Antibiotic prescribing patterns in the pediatric emergency department at Georgetown Public Hospital Corporation: a retrospective chart review

**DOI:** 10.1186/s12879-016-1512-4

**Published:** 2016-04-19

**Authors:** Suparna Sharma, Clive Bowman, Bibi Alladin-Karan, Narendra Singh

**Affiliations:** Department of Pediatrics, The Hospital for Sick Children, University of Toronto, 555 University Avenue, Toronto, Ontario M5G 1X8 Canada; Department of Pediatrics, Georgetown Public Hospital Corporation, Georgetown, Guyana; Department of Pediatrics, Humber River Regional Hospital, Toronto, Canada

**Keywords:** Antimicrobial resistance, Rational prescribing, Global health, Pediatrics

## Abstract

**Background:**

The increase in antimicrobial-resistant infections has led to significant morbidity, mortality, and healthcare costs. The impact of antimicrobial resistance is greatest on low-income countries, which face the double burden of fewer antibiotic choices and higher rates of infectious diseases. Currently, Guyana has no national policy on rational prescribing. This study aims to characterize antibiotic prescribing patterns in children discharged from the emergency department at Georgetown Public Hospital Corporation (GPHC), as per the World Health Organization (WHO) prescribing indicators.

**Methods:**

A retrospective chart review of pediatric patients (aged 1 month–13 years) seen in the GPHC emergency department between January and December 2012 was conducted. Outpatient prescriptions for eligible patients were reviewed. Patient demographics, diagnosis, and drugs prescribed were recorded. The following WHO Prescribing Indicators were calculated: i) average number of drugs prescribed per patient encounter, ii) percentage of encounters with an antibiotic prescribed, iii) percentage of antibiotics prescribed by generic name, and iv) percentage of antibiotics prescribed from essential drugs list or formulary.

**Results:**

Eight hundred eleven patient encounters were included in the study. The mean patient age was 5.55 years (*s* = 3.98 years). 59.6 % (*n* = 483) patients were male. An average of 2.5 drugs were prescribed per encounter (WHO standard is 2.0). One or more antibiotic was prescribed during 36.9 % (*n* = 299) of all encounters (WHO standard is 30 %). 90.83 % of antibiotics were prescribed from the essential drugs formulary list and 30 % of the prescriptions included the drug’s generic name. The average duration of antibiotic therapy was 5.73 days (*s* = 3.53 days). Of the 360 antibiotics prescribed, 74.7 % (*n* = 269) were broad-spectrum. B-lactam penicillins were prescribed most frequently (51.4 %), with amoxicillin being the most popular choice (33.9 %). The most common diagnoses were injuries (25.8 %), asthma (20 %), respiratory infections (19.5 %), and gastrointestinal infections (12.1 %).

**Conclusions:**

Per WHO prescribing indicators, the pediatric emergency department at GPHC has higher than standard rates of antibiotic use and polypharmacy. The department excels in adhering to the essential drug formulary. Our findings provide support for investigating drug utilization in other Guyanese settings, and to work towards developing a national rational prescribing strategy.

## Background

Antibiotics are amongst the most common medications prescribed for children [1]. Recent years, however, have seen an uncontrolled rise in antimicrobial-resistant infections, leading to increased morbidity, mortality, and healthcare costs [2]. The impact of antimicrobial resistance is arguably greatest on low-income countries, which face the double burden of fewer antibiotic choices and higher rates of infectious disease [2]. Rational prescribing practices serve to combat this global public health challenge by preventing antibiotic overuse and misuse [3]. Unfortunately, uptake of this concept has been slow, with fewer than half of all countries having any policies promoting good antimicrobial stewardship [3].

Guyana is a lower-middle-income country in South America with significant rates of communicable diseases like malaria, HIV and tuberculosis [4]. There is high potential for overuse of antimicrobials as antibiotics can be purchased without prescription and there is poor public awareness of antimicrobial resistance. Unfortunately, without a national surveillance network to monitor antimicrobial resistance, little is known about the state of antimicrobial resistance in Guyana [5]. Previous studies have shown high frequencies of methicillin-resistant S. aureus in skin and soft tissue infection isolates from Guyana, as well as multi-drug resistant M. tuberculosis, and penicillin and/or tetracycline resistance in N. gonorrhoeae isolates [6–8]. Currently, Guyana lacks a comprehensive policy on rational prescribing.

The WHO has developed prescribing indicators to detect barriers to good antimicrobial stewardship [9]. These indicators measure the performance of health care providers in prescribing drugs appropriately in primary health-care facilities, and are a commonly used standard in drug utilization studies [9]. There have been no previously published drug utilization studies measuring these indicators in a pediatric context in Guyana.

GPHC is a national referral hospital, with a 937-bed capacity making it the country’s largest hospital [10]. The GPHC emergency department sees the highest volume of pediatric outpatients in the country, with wide-ranging acuity. Health services are also provided to the public at four regional hospitals, smaller district hospitals, health centers and local health posts. In practice, patients often bypass smaller centers and present directly to regional hospitals or to GPHC for care [10]. By characterizing antibiotic prescribing patterns, as per the WHO prescribing indicators, in children discharged from the emergency department at Georgetown Public Hospital, this study aims to provide impetus for the development of a rational prescribing strategy in Guyana.

## Methods

A retrospective chart review was conducted of pediatric patients (aged 1 month–13 years) seen in the GPHC emergency department in Georgetown, Guyana. Since the treatment of infections in older teenagers can be similar to the approach used in adults, we utilized younger age cut-offs to capture pediatric antimicrobial use. The charts of all eligible patients assessed on the 1st and 15th day of each month from January to December 2012 were included. This sampling method was used to ensure a manageable sample size that was random and accounted for seasonal and temporal variation. If patient charts were missing for the 1st or 15th day of the month, charts from the next closest date were sampled instead (e.g., charts from February 16, 2012 sampled in lieu of February 15, 2012). The primary investigator (S.S.) reviewed outpatient prescriptions for these patients. Patients who absconded from hospital prior to assessment by a physician were excluded, as no prescriptions were written in these charts. For each patient encounter, the patient demographics (age, sex, race), diagnosis, and drugs prescribed were recorded. If antibiotics were prescribed, the antibiotic class, dose, route, frequency and duration of therapy was recorded. Based on this data, the following WHO Prescribing Indicators were calculated:

i) average number of drugs prescribed per patient encounter,
ii) percentage of encounters with an antibiotic prescribed,
iii) percentage of antibiotics prescribed by generic name, and
iv) percentage of antibiotics prescribed from essential drugs list or formulary.

The distribution of antibiotic prescriptions was sub-divided by class of antibiotic, spectrum of activity, and primary diagnosis. Broad-spectrum antibiotics included second to fourth generation penicillins (i.e. beta-lactam penicillins), sulfonamides, cephalosporins, fluoroquinolones and tetracycline. Narrow-spectrum antibiotics included aminoglycosides, metronidazole, macrolides and first-generation penicillins. All statistical analyses were performed using Microsoft Excel version 12.3.6. Ethical approval was obtained from the Institutional Review Board of the Ministry of Health in Guyana.

## Results & discussion

Eight hundred and fifty six charts of pediatric patients assessed in the GPHC emergency department were reviewed during the study period. Forty-five patient encounters were excluded because 44 patients absconded prior to receiving therapy and 1 patient did not fit the age criteria. Eight hundred and eleven patient encounters were included in the study. The median patient age was 5.0 years (interquartile range 2.0–9.0) and 59.6 % (*n* = 483) of patients were male (Table 1).

**Table 1:** Baseline demographics of study sample (*N* = 811)

Characteristic	Frequency	
	Number	Percentage
Age		
< 12 months	89	11.0 %
1–3 years	237	29.2 %
4–6 years	160	19.7 %
7–9 years	149	18.4 %
10–13 years	176	21.7 %
Sex		
Female	328	40.4 %
Male	483	59.6 %
Race/ethnicity		
African	468	57.7 %
Mixed	180	22.2 %
East Indian	124	15.3 %
Amerindian	21	2.6 %
Not specified	18	2.2 %

### WHO prescribing indicators

An average of 2.5 drugs were prescribed per patient encounter, as outlined in Table 2. The WHO standard for average number of drugs prescribed per patient encounter is 2.0 [11]. Rates higher than this standard are suggestive of polypharmacy, which can increase the risk of adverse drug interactions, non-adherence, and antimicrobial resistance [3]. While our observed study value is higher than the WHO index, it is similar to the values in previously published drug utilization studies. Studies of antibiotic prescriptions from pediatric emergency settings in Saudi Arabia and Jordan report an average of 2.8 and 2.4 drugs prescribed per patient encounter, respectively [12, 13]. Drug utilization studies in ambulatory pediatric settings in lower-middle income countries report a range of values for this indicator, in Sudan (2.0), Nigeria (2.6), and India (5.6) [14–16].

**Table 2:** WHO Prescribing indicators estimated from 811 prescriptions at the Georgetown Public Hospital Corporation’s pediatric emergency department in Georgetown, Guyana (*N* = 811)

WHO Prescribing Indicator	Result
Average number of drugs per encounter (*N* = 811)	2.5 (*s* = 1.54)
Percentage of encounters with one or more antibiotic prescribed (*N* = 811)	36.9 % (*n* = 299)
Percentage of antibiotics prescribed by generic name (*N* = 360)	30.0 % (*n* = 108)
Percentage of antibiotics from essential drug list (*N* = 360)	90.8 % (*n* = 327)

One or more antibiotic was prescribed during 36.9 % (*n* = 299) of all patient encounters. As a measure of antibiotic overuse, this value is close to the WHO standard, which recommends antibiotic prescriptions not exceed 30 % of patient encounters [11]. Our value fared much better in comparison to antibiotic prescription rates in pediatric settings in other developing countries like Sudan (81.3 %), Nigeria (71.1 %), and India (81.1 %) [14–16]. In higher income countries, these values ranged in pediatric settings from 18.5 % in Saudi Arabia, to 44.6 % in United Arab Emirates, and 85 % in Jordan [12, 13, 17]. Two or more antibiotics were prescribed in only 6.4 % (*n* = 52) of all encounters, suggesting that when antibiotics were prescribed in our sample, usually a single antimicrobial was used. The average duration of antibiotic therapy was 5.73 days (*s* = 3.53 days).

Ninety one percent of antibiotics were prescribed from the essential drugs formulary list. A formulary list can help aid rational prescribing by encouraging selection of medicines that are cost-effective and appropriate to local drug resistance and disease prevalence patterns. Adherence to an essential drugs list was high in our study likely because physicians had easy access to updated formularies in the emergency department. Not all of the aforementioned pediatric comparison studies reported this indicator, but of the countries that did, the percentage of drugs prescribed from a formulary varied from 60.4 % in Nigeria, to 90.2 % in India, and 100 % in United Arab Emirates [15–17].

Thirty percent of the antibiotic prescriptions in our sample used the drug’s generic name. Prescription of generics is recommended to reduce cost to patients. The use of generic agents in our sample is likely an underestimate. This is because while a brand name may be written on the prescription, the patient will often receive the drug in generic form at the dispensary due to frequent shortages of brand name agents. Since our study did not include a review of the hospital pharmacy’s dispensing records, we were unable to capture which medication the patient ultimately received. Other reasons for favoring brand name drugs may be due to concerns about quality and safety of generic products in Guyana. It may also reflect the influence of marketing by drug companies. In comparison, the percentage of drugs prescribed by generic name in pediatric settings elsewhere ranged from 2.6 % in India, to 49.3 % in Sudan, and 68.9 % in Nigeria [14–16].

### Antibiotic prescriptions

As outlined in Table 3, of the 360 antibiotics prescribed, 74.7 % (*n* = 269) were broad-spectrum. B-lactam penicillins were prescribed most frequently (51.4 %), followed by sulfonamides (13 %), cephalosporins (7.7 %) and fluoroquinolones (1.7 %). Of the B-lactam penicillins, amoxicillin was the most popular choice (33.9 %). Fixed-dose combinations of antibiotics were encountered in 12.9 % of all prescriptions. Sulfamethoxazole and trimethoprim accounted for 5.5 % of all prescriptions and was classified as a sulfonamide for the purposes of this study. Amoxicillin and clavulanic acid was classified as a beta-lactam antibiotic and accounted for 7.4 % of all prescriptions.

**Table 3:** Distribution of all antibiotics prescribed in the pediatric emergency department at GPHC (*N* = 360)

Antibiotic class	Proportion of all antibiotics prescribed (*N* = 360)
Broad spectrum agents	
B-lactam	51.4 % (*N* = 185)
Sulfonamide	13.1 % (*N* = 47)
Cephalosporin	7.8 % (*N* = 28)
Fluoroquinolone	1.7 % (*N* = 6)
Tetracycline	0.8 % (*N* = 3)
Narrow spectrum agents	
Aminoglycoside	10.6 % (*N* = 38)
Metronidazole	6.9 % (*N* = 25)
Macrolide	3.9 % (*N* = 14)
First-generation penicillins	3.9 % (*N* = 14)

### Indications for prescriptions

The most common diagnoses for which drugs were prescribed were injuries (25.8 %), asthma (20 %), respiratory infections (19.5 %), and gastrointestinal infections (12.1 %), as outlined in Table 4.

**Table 4:** Distribution of all diagnoses seen in the pediatric emergency department at GPHC, for which prescriptions were provided (*N* = 811)

Diagnosis	Frequency
Infectious Conditions	
Respiratory infections	19 % (*N* = 158)
Gastrointestinal infections	12 % (*N* = 98)
Skin, joint, bone infections	5 % (*N* = 41)
Urinary tract infections	2 % (*N* = 15)
Injuries including lacerations, bites, and burns	26 % (*N* = 209)
Asthma	20 % (*N* = 162)
Other	16 % (*N* = 128)

While it was outside the scope of this study to evaluate drug use based on rational prescribing guidelines, we reviewed the specific diagnoses for which the top three antibiotic classes were prescribed (i.e., B-lactam penicillins, sulfonamides, and cephalosporins). As outlined in Table 5, at least 73 of these broad-spectrum prescriptions were used for inappropriate indications, like asthma and viral illness. This corresponds to roughly 20 % of all the antibiotics prescribed in this study (*n* = 360). Our chart review recorded only the primary diagnosis listed for each clinical encounter, thus encounters that featured more than one diagnosis (e.g., asthma and otitis media) may be appropriate for prescribing an antibiotic. Even still, our estimate of inappropriate use is conservative and likely an underestimate as we assumed that all diagnoses that could theoretically be treated with antibiotics were clinically severe enough to warrant broad-spectrum antibiotic use at the outset, instead of a narrow spectrum agent or a wait-and-see approach. We also excluded unspecified diagnoses (e.g., fever, vomiting) and diagnoses marked as other (e.g., anemia, seizures, renal colic, etc.) from this estimate. This rough estimate of inappropriate antibiotic prescription is in line with previously published estimates, which cite 30–50 % of antibiotic use in hospitals as unnecessary or inappropriate [18].

**Table 5:** Distribution of diagnoses for which top three classes of broad-spectrum antibiotics were prescribed in the pediatric emergency department at GPHC (*N* = 260)

Diagnosis	B-lactam	Sulfonamides	Cephalosporins
	(*N* = 185)	(*N* = 47)	(*N* = 28)
Injuries	33	2	3
Pneumonia	21	0	6
Skin, joint, bone infection	21	3	2
Diarrheal disease	6^a^	21	2
Tonsillitis	19	3^a^	1
Ear infection	7	0	2
Urinary tract infection	3	7	1
Appendicitis	0	0	2
Viral illness	35^a^	5^a^	1^a^
Asthma	20^a^	1^a^	2^a^
Unspecified	18	2	1
Other	0	3	5

^a^There is no clinical evidence to support the use of this antimicrobial to treat the specified diagnosis

## Conclusions

This is the first study of antibiotic use in children in Guyana, in an outpatient context. In comparing our results to established WHO standards of drug utilization, the pediatric emergency department at GPHC has higher than standard rates of antibiotic use and polypharmacy, albeit lower than pediatric settings in other lower-middle income countries in the world. Adherence to the essential drug formulary is a strength for the department.

While it was beyond the scope of this study, our results also suggest that antibiotics are often inappropriately prescribed for non-infectious diagnoses, like asthma and viral infections. Even when used for infectious conditions, broad-spectrum antibiotics were often used as first-line therapy, which highlights the need for judicious use.

With respect to limitations, our study was retrospective in design and limited by incomplete and/or missing data. Incomplete charting on weight-based doses per child and duration and frequency of antibiotics prescribed precluded a secondary data analysis to determine if appropriate doses were prescribed for each clinical encounter. Similarly, the diagnoses associated with each encounter were at times vague (e.g., diarrhea) or unclear (e.g., not ICD-classified diagnosis), making it difficult to assess whether the recorded diagnosis accurately described the condition being treated, and whether the appropriate treatment was prescribed. Finally, with respect to generalizability, the results of this study apply only to an outpatient pediatric population in Georgetown.

Baseline data on the prevalence of antibiotic use is the first step in encouraging drug monitoring and quality improvement. As a next step, evaluating adherence of prescribing health care providers to clinical criteria for specific diagnoses will be important in assessing rational use. We hope these results provide impetus for examining drug utilization in other settings in Guyana. There is urgent need to improve surveillance of antimicrobial resistance and develop a national rational prescribing strategy in Guyana. A drug use evaluation study is the next step in helping to inform protocols for rational prescribing and antibiotic stewardship efforts at Georgetown Public Hospital.

### Availability of data and materials

The aggregate data supporting findings contained within this manuscript will be shared upon request submitted to the corresponding author. Identifying patient data will not be shared.

### Consent

As per the Institutional Review Board, written informed consent was not required from the patients involved in this study, as aggregate data was used, with no identifying personal details published.

### Ethics approval

Ethical approval was obtained from the Institutional Review Board of the Ministry of Health in Guyana. The Chief Executive Officer of the Georgetown Public Hospital Corporation granted administrative permission to access medical records.

## Abbreviations

GPHC: Georgetown Public Hospital Corporation
ICD: International Classification of Diseases
WHO: World Health Organization
